# Patient frustration with time spent on advanced ovarian and metastatic breast cancer care

**DOI:** 10.1016/j.gore.2026.102233

**Published:** 2026-10-19

**Authors:** Allison Dona, Sarah J. Boyle, Patricia Jewett, Zuofu Huang, Helen M. Parsons, Deanna Teoh, Yingling Fan, Rebecca Arend, Nicole L. Henderson, Gabrielle Rocque, Julian Wolfson, Anne Blaes, Rachel I. Vogel

**Affiliations:** a Obstetrics, Gynecology and Women's Health, University of Minnesota, 420 Delaware St SE, Minneapolis, MN, 55455, USA; b Division of Environmental Health Sciences, University of Minnesota, 420 Delaware St SE, Minneapolis, MN, 55455, USA; c Division of Biostatistics and Health Data Science, University of Minnesota, 2221 University Ave SE, Suite 200, Minneapolis, MN, 55414, USA; d Department of Statistics, Grinnell College, 1116 8th Ave, Grinnell, IA, 50112, USA; e Masonic Cancer Center, University of Minnesota, 420 Delaware St SE, Minneapolis, MN, 55455, USA; f Division of Health Policy and Management, University of Minnesota, 420 Delaware St SE, Minneapolis, MN, 55455, USA; g Humphrey School of Public Affairs, University of Minnesota, 301 19th Ave S, Minneapolis, MN, 55455, USA; h Division of Gynecologic Oncology, University of Alabama at Birmingham, O'Neal Comprehensive Cancer Center, 1700 6th Ave S, Birmingham, AL, 35233, USA; i Divisions of Hematology & Oncology and Gerontology, Geriatrics, & Palliative Care, University of Alabama at Birmingham, O'Neal Comprehensive Cancer Center, 2000 6th Ave S, Birmingham, AL, 35233, USA; j Division of Hematology, Oncology, and Transplantation, University of Minnesota, 420 Delaware St SE, Minneapolis, MN, 55455, USA

**Keywords:** Time burden, Survivorship, Frustration, Cancer care

## Abstract

**Objective:**

While cancer treatment requires a significant time commitment, the objective time spent does not necessarily reflect a patient's perceived time burden. To understand which specific aspects of care are most burdensome, we sought to quantify frustration with time spent on various cancer care-related tasks among individuals receiving treatment for advanced ovarian or metastatic breast cancer.

**Methods:**

Upon enrolling in a longitudinal study, 83 participants completed a baseline survey regarding frustration with time spent on cancer care tasks. Frustration was measured on a scale of 0 (not at all frustrating) to 10 (extremely frustrating) for cancer care-related activities including travel, waiting, appointments, and logistical tasks. Scores were compared by employment status, dependent status, and distance from the cancer clinic using Wilcoxon rank-sum and Kruskal-Wallis tests.

**Results:**

Median frustration scores for most tasks were low, but ranges were wide. Activities associated with the greatest frustration included number of trips to the cancer center, travel time per trip, wait times, and paperwork. Participants receiving disability benefits reported high frustration with nearly all tasks. Additionally, participants with dependents reported greater frustration with the number of trips, paperwork, time off work, and organizing dependent care. Participants living 30+ miles from the cancer center reported more frustration with travel time per trip and arranging transportation.

**Conclusion:**

Time-related frustrations in cancer care are not uniform and more heavily impact participants who are on disability, have dependents, or experience geographic barriers. Individualized care coordination and optimized systemic workflows may minimize care-related time burden for these vulnerable populations.

## Highlights

• Frustration with time spent on cancer care varies widely among cancer survivors.
• Patients on disability reported higher frustration across nearly all tasks.
• Having dependents amplified frustration with clinic trips and time off work.
• Those living further from clinic had higher travel and transportation frustration.
• Time spent and experienced burden are both important for understanding time burden.

## 1 Introduction

The time burden of cancer care is a newly recognized dimension of treatment toxicity and an emerging area of research. We define this burden as time spent on cancer care and associated activities (e.g., travel, waiting for appointments) that actively interferes with a patient's life responsibilities and well-being (Fundytus et al., 2021; Gupta et al., 2022; Yabroff et al., 2014; Gupta et al., 2023a). Although standardized methods have not yet been established, studies have started to attempt to measure time burden, predominantly by quantifying the objective time spent on different areas of care (Vogel et al., 2025; Rocque et al., 2020; Waks et al., 2025; Quinn et al., 2024; Gupta et al., 2023b; Kagalwalla et al., 2024). While these objective measurements are important components of time burden, not all time spent on cancer care is viewed as burdensome by patients; therefore, objective time spent does not directly translate to experienced burden (Gupta et al., 2024). Investigating the subjective experience is crucial to capturing the true impact of these care-related disruptions on patients and their families.

To effectively address these disruptions, we must first understand which specific care tasks drive patient frustration and identify the populations most vulnerable to their impact. Qualitative work has shown that individuals with cancer experience burdens related to travel, appointment schedules, healthcare system navigation, and relationship consequences; however, it remains unclear how much of this burden is driven by the amount of time spent versus other overlapping stressors, such as logistic or administrative hurdles (Dona et al., 2024). In addition, system- and individual-level factors influence the amount of burden experienced from cancer care. Patients with competing life demands – such as financial barriers or caregiving responsibilities – may be the most negatively impacted when cancer care disrupts their limited time (Dona et al., 2024).

Consequently, this study sought to describe patient-reported frustration with time spent on cancer care tasks among individuals receiving treatment for advanced ovarian or metastatic breast cancer and to explore how these frustrations vary by employment status, dependent status, and distance from the cancer clinic.

## 2 Methods

### 2.1 Study design and population

We conducted a prospective cohort study of individuals diagnosed with and receiving treatment for advanced stage (stage III or IV) ovarian, fallopian tube, or primary peritoneal cancer or metastatic breast cancer. The goal of the overall study was to collect detailed quantitative data regarding time use using a mobile application to describe the time burdens of cancer care; details provided elsewhere (Vogel et al., 2025). Briefly, participants were asked to complete baseline and follow-up surveys and use the mobile application to track time use for 28 days. The study was approved by the University of Minnesota Institutional Review Board (STUDY00016834) and registered with ClinicalTrials.gov (NCT05708703). This analysis utilized cross-sectional data from the baseline survey only.

Participants were recruited in person during cancer clinic visits at the University of Minnesota and the University of Alabama at Birmingham from December 2023 to September 2024. Eligible participants were 18 years or older, receiving anti-cancer treatment at the time of study entry, and able to read and write in English. All participants provided written informed consent.

### 2.2 Measures

The primary outcomes of interest for this analysis were questions created by the study team to assess patient-reported frustration with time spent on various cancer care-related tasks and activities. Participants were asked to rate the level of frustration experienced with time spent on each activity on a scale of 0 (not at all frustrating) to 10 (extremely frustrating). The activities included trips to the cancer center (number of and travel time per trip), arranging transportation, wait times (at lab, waiting for provider, at infusion center, at radiation center, and other wait time), time spent with providers, managing paperwork (health or disability insurance, bills, pre-authorizations, etc.), and care coordination (making appointments, coordinating multiple healthcare visits, interacting with pharmacy, having to take time off work, and having to organize care for dependents to attend appointments). Participants were able to add and rate additional frustrating free-text activities.

The primary exposures of interest were employment status, dependent status, and distance between home and the cancer clinic. Participants self-reported their current employment status and were categorized as working (full-time/part-time/self-employed/student), not employed and receiving disability benefits, or not working (otherwise not working/retired). Individuals receiving disability were categorized separately from other non-working or retired individuals to account for the distinct socioeconomic vulnerabilities and systemic interactions unique to this population. For dependent status, we asked participants if they have any dependents, defined as persons they take care of and/or support financially regardless of dependent's age, and categorized as yes vs. no. Distance between their home and the cancer clinic was calculated based on geodetic distance in miles between the centroid of the zip code of their residence and zip code of their respective main cancer clinic and categorized as less than 30 miles vs. 30 or more miles from the cancer clinic.

### 2.3 Statistical analysis

Demographic and clinical data were summarized with descriptive statistics. Frustration levels with each activity were summarized using medians and inter-quartile ranges (IQR) and compared between groups using non-parametric methods, including Wilcoxon rank-sum and Kruskal-Wallis tests. A sensitivity analysis was performed to evaluate cancer type (metastatic breast vs. advanced ovarian cancer) as a potential modifier using interaction models and stratified analyses. Data were analyzed using SAS 9.4 (Cary, NC) and figures were created using R (version 4.5.2).

## 3 Results

Of the 100 individuals approached to participate, 89 (89%) enrolled in the study and 83 (93%) completed the baseline survey and were included in this analysis. The median age of participants was 59 years (range: 23–79). All participants identified as female; 19% identified as Black, 76% as White, 2% as mixed race, and 2% as another race; 5% identified as Hispanic (Table 1). Just under half (41%) were employed, and 35% reported having dependents. About half (48%) lived within 30 miles of the cancer clinic. There was a similar number of participants diagnosed with advanced ovarian (*n* = 43, 51.8%) and metastatic breast cancer (*n* = 40, 48.2%). Participants were most often receiving treatment for recurrence or disease progression (57%); the remaining were receiving frontline (30%) or maintenance therapy (13%). The median time since diagnosis was 2 (range: 0–11) years.

**Table 1:** Study participant demographic and clinical characteristics (*n* = 83).

Characteristic	Median (range)	
**Age, years**	58.5 (22.8–78.5)	
**Time since diagnosis, years**	1.8 (0.1–11.0)	
	**n**	**%**
**Gender**		
Female	83	100.0
**Race**		
Black	16	19.3
White	63	75.9
More than 1 race	2	2.4
Other	2	2.4
**Ethnicity**		
Hispanic	4	4.8
Non-Hispanic	76	91.6
Unknown	3	3.6
**Cancer type**		
Metastatic breast cancer	40	48.2
Advanced stage ovarian cancer	43	51.8
**Cancer type**		
Front-line / upfront treatment	25	30.1
Maintenance	11	13.3
Treatment for recurrent/progressive disease	47	56.6
**Household Annual Income**		
<$50,000	31	37.4
$50,000–99,999	22	26.5
$100,000–149,999	8	9.6
≥$150,000	15	18.1
Prefer not to answer	7	8.4
**Partner status**		
Not partnered	34	41.0
Partnered	49	59.0
**Dependent Status**		
Dependents	29	34.9
No Dependents	54	65.1
**Clinic**		
University of Alabama – Birmingham	44	53.0
University of Minnesota	39	47.0
**Distance home from clinic**		
<30 miles	40	48.2
30+ miles	43	51.8
**Employment Status**		
Disability	14	16.9
Work full/part time, student	34	41.0
Not working	31	37.4
*Missing*	4	4.8

The median reported frustration with most cancer care tasks was low, but the range was wide for all tasks (Table 2). The activities associated with the greatest frustration were the number of trips to the cancer center, travel time per trip, wait times, and paperwork. Other activities reported as frustrating by participants in free-text responses included planning travel, driving long distances, waiting for messages from the provider or scheduler, appointments exceeding stated times, appointments cutting into leisure activities, waiting for appointments to be scheduled, and waiting for test results.

**Table 2:** Self-reported frustration (0–10) across cancer care activities among individuals receiving treatment for advanced stage ovarian or metastatic breast cancer (*n* = 83).

Variable	n	Median (Q1-Q3)	Range
Travel-related Tasks			
Number of trips needed for cancer care	82	2.5 (1–5)	0–10
Travel time per trip	82	2.0 (1–5)	0–10
Finding means of transportation	83	1.0 (0–4)	0–10

Clinic-related Tasks			
Wait time	82	1.0 (0–4)	0–10
At lab	82	1.0 (0–4)	0–10
Waiting for provider	83	1.0 (0–3)	0–10
At infusion center	82	1.0 (0–4)	0–10
At radiation center	47	0.0 (0–1)	0–8
Other wait time	53	0.0 (0–1)	0–7
Time spent with providers	83	0.0 (0–2)	0–10

Logistical Tasks			
Paperwork (health or disability insurance, bills, pre-authorizations, etc.)	81	2.0 (0–4)	0–10
Care coordination	82	1.0 (0–2)	0–10
Making appointments	82	1.0 (0–2)	0–10
Coordinating multiple healthcare visits	83	1.0 (0–3)	0–9
Interactions with pharmacy	81	1.0 (0–2)	0–10
Having to take time off work	61	0.0 (0–4)	0–9
Having to organize care for dependents	66	0.0 (0–2)	0–10

Differences in frustration were observed for nearly all clinical, administrative, and logistical tasks among those on disability compared to those who were employed or otherwise not working/retired (Fig. 1). There were notable differences in frustration regarding travel time (median 6 [Q1-Q3: 5–7] vs. 1 [Q1-Q3: 0–3.75] vs. 1 [Q1-Q3: 0–3], *p* < 0.0001), wait times (6 [4–8] vs. 2 [0–4.5] vs. 1 [0–2], *p* = 0.0008), and care coordination (3 [2–4] vs. 1 [0–2] vs. 0 [0–1.75], *p* = 0.002) for participants who were on disability, retired/non-working, and employed respectively. Employed participants reported sources of frustration similar to those not working or retired. There were no differences in the conclusions when stratified by cancer type.

**Fig. 1:**
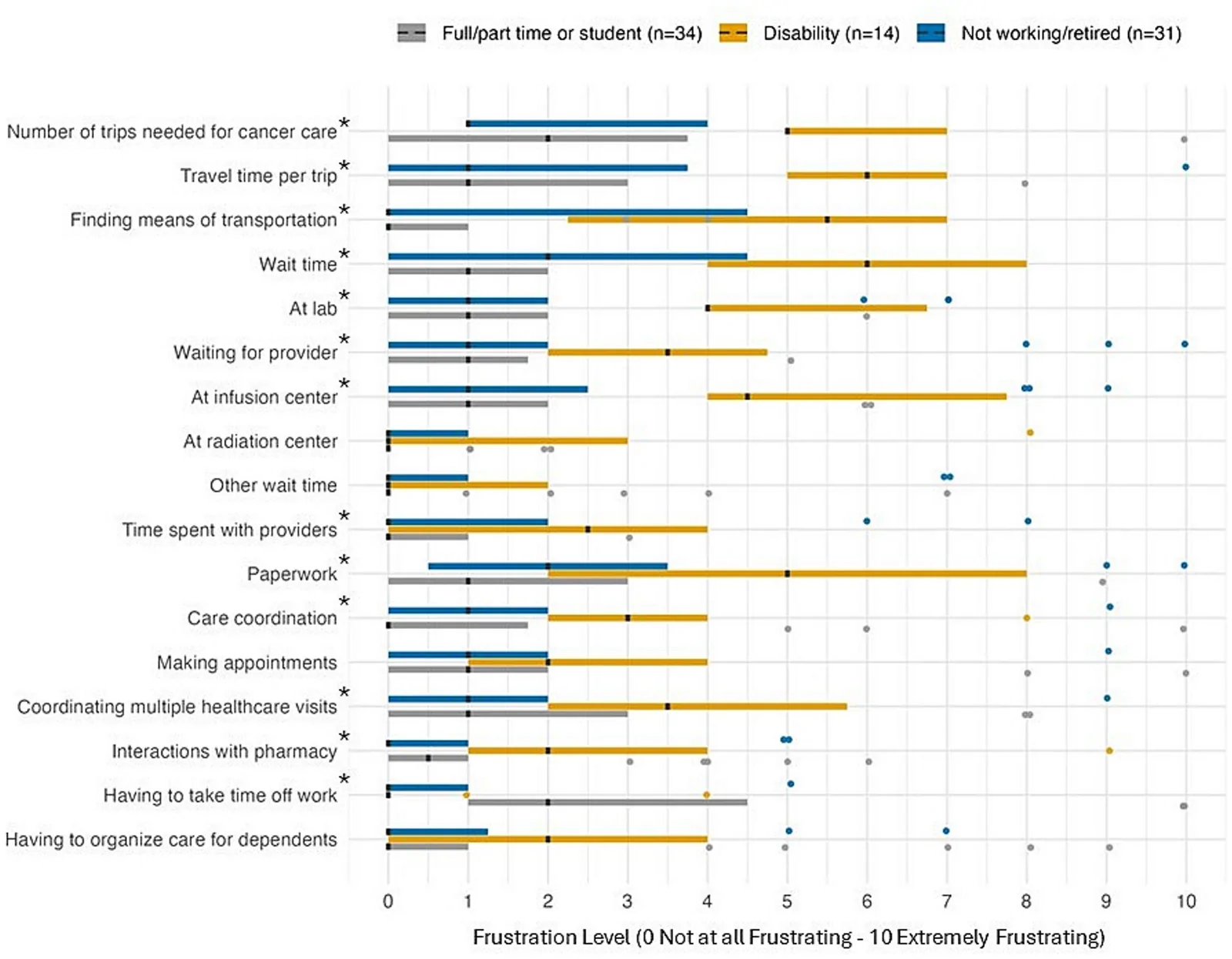
Distributions of self-reported frustration (0–10) across cancer care activities among individuals receiving treatment for advanced stage ovarian or metastatic breast cancer by employment status. Vertical lines within the boxes represent the median, box boundaries indicate the 25th and 75th percentiles. Outlier data points are integers, jittered for visibility. Asterisk (*) indicates Kruskal-Wallis test *p* < 0.05.

Participants with dependents, compared to those without, reported greater frustration with the number of clinic trips (4 [2–7] vs. 2 [1–4], *p* = 0.02), taking time off work (2 [0–4] vs. 0 [0–2], *p* = 0.03), and arranging care for family or pets (1.5 [0–5] vs. 0 [0–1], *p* = 0.004; Supplemental Fig. 1). Participants living 30 or more miles away experienced greater frustration with travel time per trip (4 [1–6] vs. 1 [0–3], *p* = 0.004) and arranging transportation (1 [0–6] vs. 0 [0–1.5], *p* = 0.01; Supplemental Fig. 2). The results were similar when stratified by cancer type for both dependent status and distance from the cancer clinic.

## 4 Discussion

Our study demonstrates that while median levels of time-related frustration are generally low, wide variability exists among patients. Our findings reinforce the concept that objective time spent on cancer care does not directly parallel subjective time burden (Gupta et al., 2024). The most frustrating tasks were administrative tasks such as managing paperwork and needing to take multiple trips to the cancer center, and we identified differences in reported frustration by employment status, having dependents, and distance between home and the cancer center.

A key finding is that patients receiving disability benefits are particularly vulnerable to time burden. This cohort reported disproportionately higher frustration across nearly all tasks compared to both employed and retired individuals. The causes of higher frustration reported by this group are likely multifactorial. Cancer and its treatment can interfere with one's ability to work due to time and symptom burdens, and those with greater health burdens are more likely to experience work disability (Mahumud et al., 2020). The increased health burdens in this group likely contribute to increased time spent on care and subsequent frustration. In addition, work disability can have significant downstream economic and quality of life impacts which could contribute to greater experienced time burden. While additional research is needed to fully understand the drivers of time burden, patients on disability represent a high-priority population for targeted, intensive administrative navigation support.

Our findings expand upon the prior work by Dona et al., who explored time and logistical priorities among breast and gynecologic cancer survivors (Dona et al., 2025). While both studies underscore the impact of dependents and the shared desire to minimize clinic trips, our findings diverge regarding employment and geography. Dona et al. observed that employed patients prioritized minimizing travel and wait times more than retired patients; however, our quantitative frustration scores were similar between these two groups except for frustration of taking time off work. Conversely, we found that participants living farther from the cancer center reported greater frustration with certain tasks, while Dona et al. did not find differences in the importance of minimizing time by rural-urban status (Dona et al., 2025). Participants with dependents reported greater frustration with certain tasks and were more likely to report minimizing time as important compared to those without dependents in both studies, although this finding was not statistically significant in Dona et al.'s study (Dona et al., 2025). These differences may reflect the clinical differences in our study populations: while Dona et al. surveyed a broad survivor cohort, our sample exclusively evaluated patients undergoing active treatment for advanced or metastatic disease, where the persistent clinical demand may amplify burdens.

To design effective clinic- and system-level interventions, future research must disentangle time expenditures from the specific structural mechanisms driving patient distress. For instance, frequent trips to a cancer center impose both a time penalty (hours lost) and a logistical penalty (arranging transit, balancing schedules). Clinic workflows may be able to mitigate these distinct pressures. For example, time burden can be reduced by structural shifts such as telehealth utilization, localized laboratory testing, and clustering of multi-disciplinary appointments. Logistical distress can be addressed via community partnerships to help provide transportation and dedicated caseworkers to manage insurance paperwork. To address the wide variability in patient frustration and advance this research area, future studies must evaluate objective time spent and subjective patient perception concurrently. Ongoing work is assessing how prospective time-tracking tools paired with real-time patient-reported outcomes can capture precise duration (e.g., travel minutes, waiting room delay) alongside immediate emotional responses (e.g., frustration, distress). Future work needs to uncover how these time demands and experiences change during the disease trajectory, including periods of intense therapy and surveillance. Further, mixed-methods interventional trials are needed to evaluate whether targeted structural modifications, such as appointment clustering or dedicated administrative navigation, effectively reduce objective time burdens and translate into meaningful reductions in patient-reported frustration, particularly among highly impacted subgroups like patients receiving disability benefits or managing dependent care.

While this study benefits from the inclusion of a diverse group of individuals receiving treatment for ovarian and breast cancer, this study has several limitations. First, while stratification by cancer type did not change our conclusions, there may be key clinical differences that could impact the time spent and subsequent frustration such as the specific treatment regimen that we were unable to capture due to sample size constraints. Second, the mobile application was available only in English, excluding non-English-speaking populations who likely navigate even steeper systemic and logistical hurdles. Third, our sample size constrained our ability to evaluate more subtle, intersecting socioeconomic determinants of time burden. Finally, selection bias may be present; individuals experiencing increased burden due to their cancer care may have been less likely to enroll in the study.

## 5 Conclusion

These findings highlight the necessity of distinguishing between the objective time associated with cancer care and a patient's subjective perception of time burden, and both should be measured concurrently to further identify the relationship between time and burden in future studies. By identifying differences in frustration by employment, disability, and dependent care, this work provides a foundational framework for future care-delivery interventions aimed at minimizing time burdens of cancer care and individualization of care coordination to preserve quality of life for individuals with cancer.

## Ethics approval and patient consent

This study was approved by the University of Minnesota Institutional Review Board, and all participants provided written informed consent.

## Studies in humans

This study was performed in compliance with relevant laws, regulatory frameworks, and guidelines where the research took place.

This study was approved by the University of Minnesota Institutional Review Board.

(Approval No. STUDY00016834)

## Clinical trials

The results of this clinical trial and any associated work have been posted in a registry.

This clinical trial was registered with number NCT05708703 (Clinicaltrials.gov).

## CRediT authorship contribution statement

**Allison Dona:** Writing – original draft, Methodology. **Sarah J. Boyle:** Writing – original draft, Methodology. **Patricia Jewett:** Writing – review & editing, Methodology, Conceptualization. **Zuofu Huang:** Writing – review & editing, Formal analysis. **Helen M. Parsons:** Writing – review & editing, Methodology, Conceptualization. **Deanna Teoh:** Writing – review & editing, Conceptualization. **Yingling Fan:** Writing – review & editing, Methodology, Conceptualization. **Rebecca Arend:** Writing – review & editing, Methodology, Conceptualization. **Nicole L. Henderson:** Writing – review & editing, Methodology. **Gabrielle Rocque:** Writing – review & editing, Supervision, Methodology, Conceptualization. **Julian Wolfson:** Writing – review & editing, Supervision, Methodology, Formal analysis, Conceptualization. **Anne Blaes:** Writing – review & editing, Methodology, Conceptualization. **Rachel I. Vogel:** Writing – review & editing, Supervision, Methodology, Funding acquisition, Conceptualization.

## Informed consent and patient details

Written informed consent to take part in the study and to publish the article has been obtained from all participants or their legal representatives. The privacy rights of participants have been observed.

## Funding

This study was supported by a National Institutes of Health National Cancer Institute grant (1R01CA277714) to RIV and JW. ACD is supported by NIH F30 Award 1F30MD019959–01 and University of Minnesota MSTP funding NIH T32 GM156735. RIV, HP and AB are supported in part by grant P30 CA77598 from the National Institutes of Health to the Masonic Cancer Center, University of Minnesota. RA and GR are supported in part by grant P30 CA013148 from the National Institutes of Health to the O'Neal Comprehensive Cancer Center. Use of REDCap is supported by UM1TR004405 from the National Institutes of Health‘s National Center for Advancing Translational Sciences.

## Declaration of competing interest

The authors declare the following financial interests/personal relationships which may be considered as potential competing interests: Rachel I. Vogel & Julian Wolfson report financial support was provided by National Institutes of Health National Cancer Institute. Allison Dona, Rachel I. Vogel, Helen Parsons, Anne Blaes, Rebecca Arend, & Gabrielle Rocque report financial support was provided by National Institutes of Health. Yingling Fan & Julian Wolfson report a relationship with Daynamica, Inc that includes: board membership and equity or stocks. Yingling Fan & Julian Wolfson have a patent issued to Regents of the University of Minnesota. If there are other authors, they declare that they have no known competing financial interests or personal relationships that could have appeared to influence the work reported in this paper.

## Figures and Tables

**Supplemental Fig. 1:**
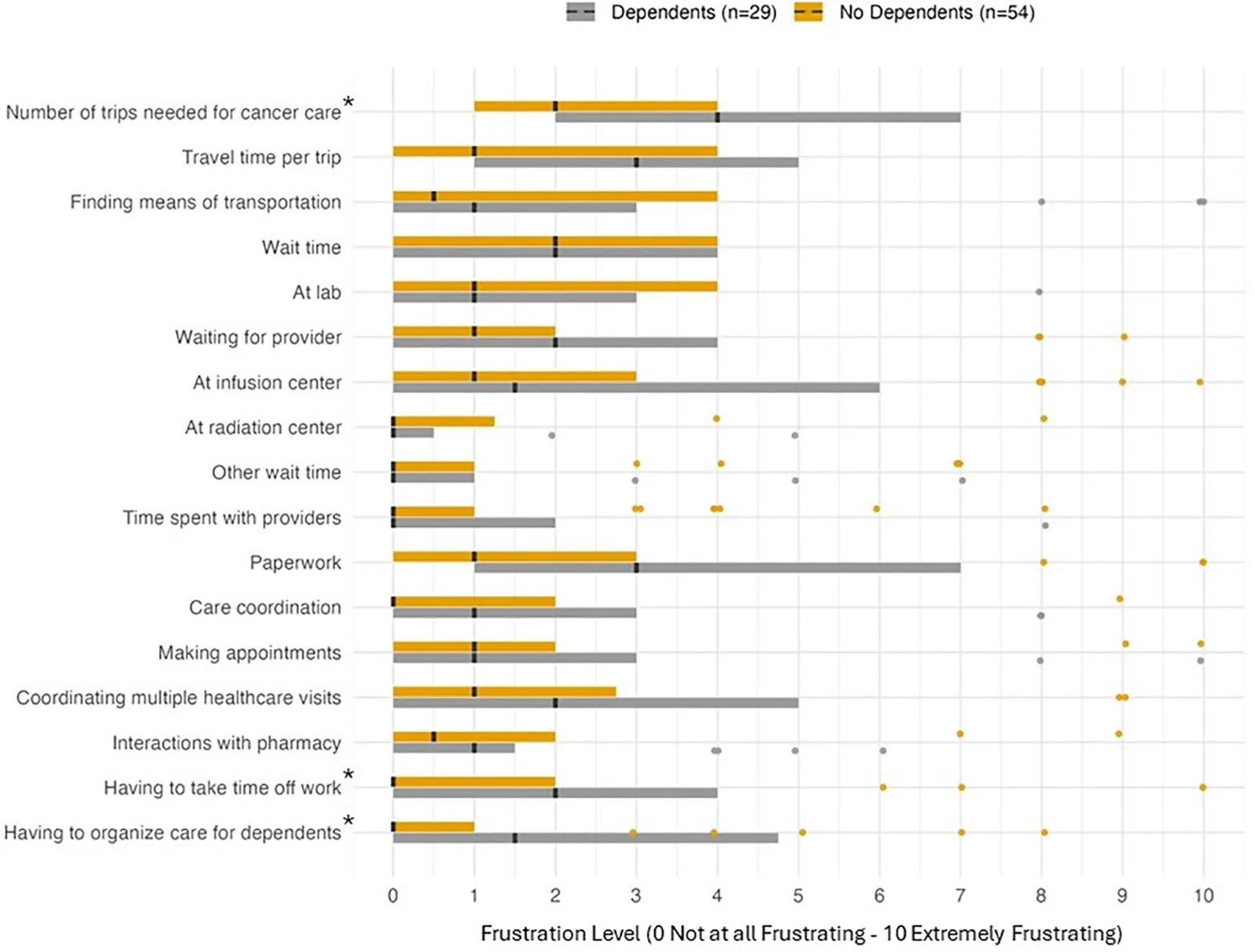
Distributions of self-reported frustration (0−10) across cancer care activities among individuals receiving treatment for metastatic breast and advanced stage ovarian cancer by dependent status. Vertical lines within the boxes represent the median, box boundaries indicate the 25th and 75th percentiles. Outlier data points are integers, jittered for visibility. Asterisk (*) indicates Wilcoxon rank-sum test *p* < 0.05.

**Supplemental Fig. 2:**
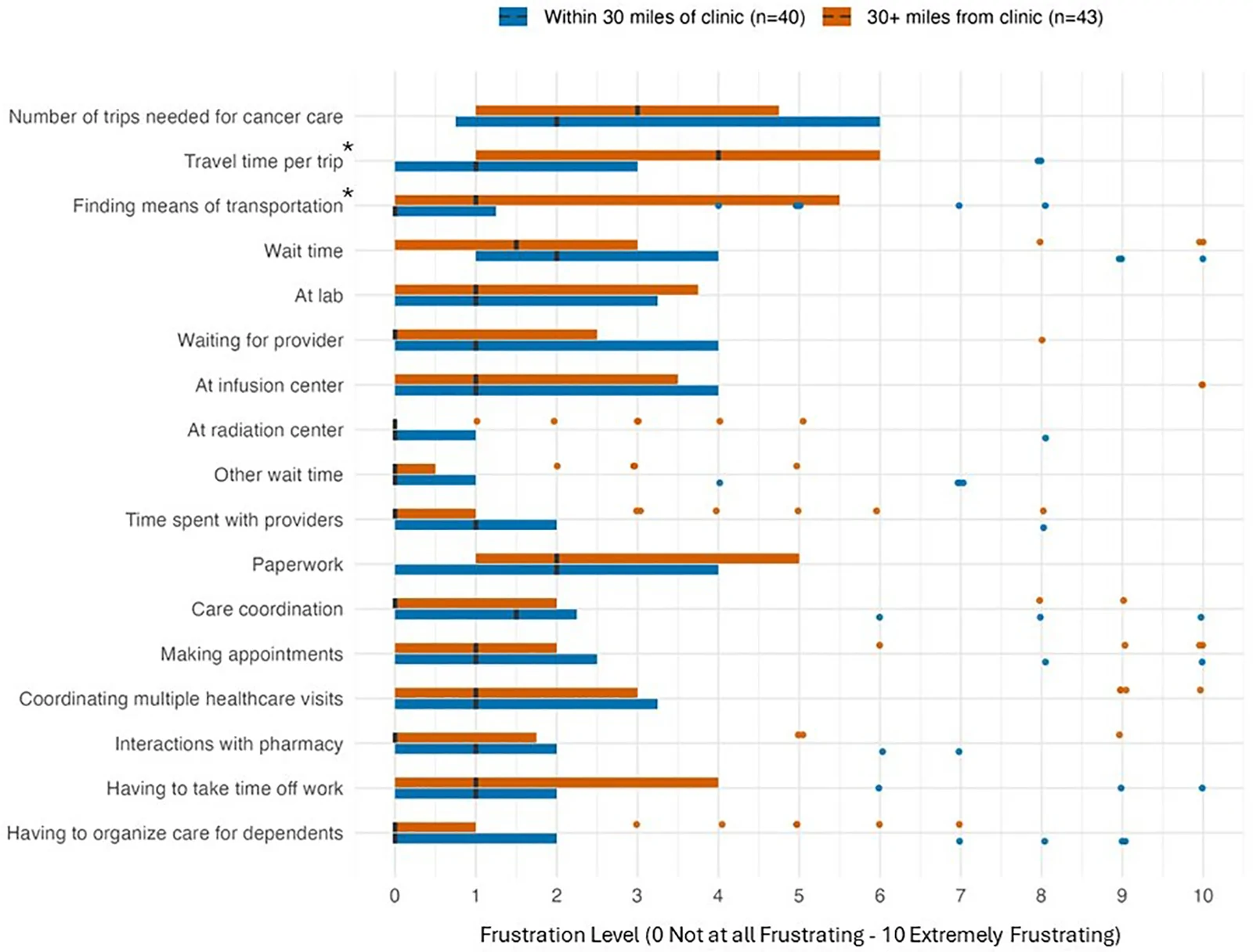
Distributions of self-reported frustration (0–10) across cancer care activities among individuals for receiving treatment metastatic breast and advanced stage ovarian cancer by distance from the cancer clinic. Vertical lines within the boxes represent the median, box boundaries indicate the 25th and 75th percentiles. Outlier data points are integers, jittered for visibility. Asterisk (*) indicates Wilcoxon rank-sum test *p* < 0.05.

## Data Availability

De-identified survey data collected from the baseline survey will be made publicly available upon publication.
